# Single-Cell Transcriptomics Uncovers Cellular Heterogeneity, Mechanisms, and Therapeutic Targets for Parkinson’s Disease

**DOI:** 10.3389/fgene.2022.686739

**Published:** 2022-05-04

**Authors:** Jianjun Huang, Li Liu, Lingling Qin, Hehua Huang, Xue Li

**Affiliations:** ^1^ Department of Neurology, Youjiang Medical University for Nationalities, Affiliated Hospital of Youjiang Medical University for Nationalities, Baise, China; ^2^ Department of Cardiology, Youjiang Medical University for Nationalities, Affiliated Hospital of Youjiang Medical University for Nationalities, Baise, China; ^3^ Department of Medical Quality Management, Affiliated Hospital of Youjiang Medical University for Nationalities, Baise, China; ^4^ Department of Anatomy, Youjiang Medical University for Nationalities, Baise, China; ^5^ Department of Electrophysiology, Affiliated Hospital of Youjiang Medical University for Nationalities, Baise, China

**Keywords:** Parkinson’s disease, single-cell RNA sequencing, cellular heterogeneity, molecular mechanisms, therapeutic targets

## Abstract

**Objective:** This study aimed to exploit cellular heterogeneity for revealing mechanisms and identifying therapeutic targets for Parkinson’s disease (PD) *via* single-cell transcriptomics.

**Methods:** Single-cell RNA sequencing (scRNA-seq) data on midbrain specimens from PD and healthy individuals were obtained from the GSE157783 dataset. After quality control and preprocessing, the principal component analysis (PCA) was presented. Cells were clustered with the Seurat package. Cell clusters were labeled by matching marker genes and annotations of the brain in the CellMarker database. The ligand–receptor networks were established, and the core cell cluster was selected. Biological functions of differentially expressed genes in core cell clusters were analyzed. Upregulated marker genes were identified between PD and healthy individuals, which were measured in 18 PD patients’ and 18 healthy individuals’ blood specimens through RT-qPCR and Western blotting.

**Results:** The first nine PCs were determined, which can better represent the overall change. Five cell clusters were identified, including oligodendrocytes, astrocytes, neurons, microglial cells, and endothelial cells. Among them, endothelial cells were the core cell cluster in the ligand–receptor network. Marker genes of endothelial cells possessed various biological functions. Among them, five marker genes (*ANGPT2*, *APOD*, *HSP90AA1*, *HSPA1A*, and *PDE1C*) were upregulated in PD patients’ than in healthy individuals’ endothelial cells, which were confirmed in PD patients’ than in healthy individuals’ blood specimens.

**Conclusion:** Our findings revealed that the cellular heterogeneity of PD and endothelial cells could play a major role in cell-to-cell communications. Five upregulated marker genes of endothelial cells could be underlying therapeutic targets of PD, which deserve more in-depth clinical research.

## Introduction

Parkinson’s disease (PD) is a neurodegenerative movement disorder with insidious onset, which affects 1%–2% of older people over 65 years (Chang et al., 2019). It possesses distinct pathological characteristics of progressive degeneration of midbrain-type dopaminergic neurons in the substantia nigra (Hanan et al., 2020). Patients usually present typical dyskinesias such as stiffness, tremors, and bradykinesia and non-motor disorders such as cognitive and sensory disorders (Ásgrímsdóttir and Arenas, 2020). Despite extensive research on the mechanisms of PD, its pathogenesis still needs more profound elucidation. The current treatment options are symptomatic, which cannot solve the root causes of PD or restrain the loss of midbrain-type dopaminergic neurons (Ásgrímsdóttir and Arenas, 2020). With the progression of PD, these therapies inevitably lose their effectiveness and have side effects. Thus, it highlights the critical significance for novel therapy aimed at the root causes of PD.

Traditional high-throughput sequencing technology needs to obtain enough DNA samples from many cells, so sequencing data are the integral characteristic information of these cells. The single-cell RNA sequencing (scRNA-seq) technology is a high-throughput sequencing analysis of the genome and transcriptome at the single-cell level, which can reveal the gene structure and gene expression dynamics of a single cell and reflect the heterogeneity between cells (González-Silva et al., 2020). For example, Katarína Tiklová et al. applied scRNA-seq to identify graft compositions derived from stem cells in a PD model, addressing an outstanding issue in cell replacement (Tiklová et al., 2020). Furthermore, Charmaine Lang et al. reconstructed PD development and corrected Parkinson’s cellular phenotypes *via* scRNA-seq of dopamine neurons derived from induced pluripotent stem cells (Lang et al., 2019). Hugo J R Fernandes et al. uncovered specific stress responses in dopamine neurons of PD based on single-cell transcriptomics (Fernandes et al., 2020). These studies emphasize the importance of scRNA-seq in the understanding of PD progression and the development of cell replacement therapies. Herein, this study applied scRNA-seq profiling of the midbrain specimens from PD and healthy individuals to exploit the cellular heterogeneity of PD, which offered novel insights into the mechanisms and therapeutic targets for this disease.

## Materials and Methods

### scRNA-Seq Data

scRNA-seq data of midbrain specimens from five idiopathic PD patients and six healthy individuals were downloaded from the Gene Expression Omnibus (GEO) database (https://www.ncbi.nlm.nih.gov/gds/; accession: GSE157783) (Badanjak et al., 2021). Single-nucleus suspension was prepared by scraping off the tissues from the glass slides and utilizing the modified version of the standard 10x Genomics nuclei isolation protocol. Single-nuclei barcoded cDNA library was prepared according to the 10x Genomics Chromium system and sequenced on the NovaSeq 6000 Illumina platform.

### Quality Control and Preprocessing

First, empty droplets that did not have any cells were identified, and barcode-swapped pseudo-cells were then removed utilizing the DropletUtils package in R (version 1.10.2) (Griffiths et al., 2018; Lun et al., 2019). The total unique molecular identifier (UMI) count, the number of cells containing the count, and the percentage of the count were separately calculated *via* the calculateQCMetrics function in the scater package (McCarthy et al., 2017). Low-quality cells were removed based on the criteria of the proportion of mitochondrial genes ≤ 10% and ribosomal genes ≥10%. Mitochondrial and ribosomal genes often consume a large fraction of reads in the scRNA-seq data, and their relative abundance varies from sample to sample (Jaeger et al., 2021). The aforementioned genes were not interesting to this study, which were removed for downstream analyses. Afterward, the average count of genes between all cells, the percentage of cells that were non-zero for each gene, and the percentage of a subset of cells to the total cells based on the average count were calculated separately *via* the perFeatureQCMetric function. Using the NormalizeData function in the Seurat package (Butler et al., 2018), the expression matrix of each sample after filtering was standardized with the LogNormalize method.

### Principal Component Analysis

The top 2000 genes with the largest variation among cells were screened utilizing the runPCA function. Then, with these 2000 genes as the input, the ScaleData function in the Seurat package was utilized to linearly scale the expression data. The linear dimensionality reduction analysis (PCA) was presented *via* the runPCA function in the Seurat package, which was visualized by the VizDimLoadings function. After dimensionality reduction, the first two principal components (PCs) were extracted to draw a scatter plot with the DimPlot function. The PCs were ranked based on the percentage of variance by each PC, and the optimal number of PCs was determined. Using the DimHeatmap function, the top 30 genes were depicted for each PC.

### Cell Cluster

Batch effects were removed by the ScaleData function of the Seurat package. After selecting the top PCs with the largest standard deviation, the FindNeighbors and FindClusters functions in the Seurat package were utilized to perform the cell cluster analysis. Then, UMAP was presented *via* the RunUMAP function in the Seurat package.

### Cluster Marker Genes

Marker genes between each cluster and all other cells were calculated *via* the FindAllMarkers function in the Seurat package with the cutoff of |log fold change (FC)| ≥ 0.1, the expression ratio of the cell population ≥0.25, and *p* value ≤0.05. In accordance with the existing annotations of the brain in the CellMarker database (http://biocc.hrbmu.edu.cn/CellMarker/or http://bio-bigdata.hrbmu.edu.cn/CellMarker/), the cells were labeled (Zhang et al., 2019).

### Pseudotime Analysis

The Monocle 3 package in R was utilized to perform the pseudotime analysis of each cell type (Qiu et al., 2017). Genes that were expressed in at least 5% of cells were selected. The dimensionality reduction analysis was presented *via* the reduceDimension function, and cells were clustered by the clusterCells function. Afterward, the genes that were different between cell clusters (*p* value <0.05) were determined using the differentialGeneTest function. Using the reduceDimension function, the dimensionality reduction analysis of the cells was carried out based on these differential genes with the Discriminative Dimensionality Reduction *via* learning a Tree (DDRTree) method. Finally, the cells were sorted and visualized using the orderCells function.

### Ligand–Receptor-Mediated Multicellular Signal

In accordance with the ligand–receptor pairing data from a previous study (Ramilowski et al., 2015), the receptor–ligand pairs were analyzed according to marker genes in various cell clusters. A ligand–receptor network was then constructed *via* the Cytoscape software (Doncheva et al., 2019). Moreover, the receptor–ligand pair genes were extracted. The core cell cluster with the largest number of intercellular receptor–ligand pairs was selected.

### Differential Expression Analysis

Differentially expressed genes (DEGs) in the core cell cluster between PD patients and healthy individuals were screened with the cutoff of |FC| > 1.5 and adjusted *p* value <0.05.

### Functional Enrichment Analysis

Gene Ontology (GO) of marker genes was presented based on the Database for Annotation, Visualization, and Integrated Discovery (DAVID; http://www.david.niaid.nih.gov) (Dennis et al., 2003). GO contains three classifications: the biological process (BP), cellular component (CC), and molecular function (MF). Moreover, the Kyoto Encyclopedia of Genes and Genomes (KEGG) enrichment analysis was used to probe underlying pathways enriched by marker genes.

### Protein–Protein Interaction

Functional and physical interactions of DEGs in endothelial cells were analyzed by the STRING database (http://string-db.org/) (Szklarczyk et al., 2017). A PPI network was visualized *via* Cytoscape software. The degree of each node was calculated.

### Patients and Specimens

This study enrolled 18 PD patients and 18 healthy participants from the Affiliated Hospital of Youjiang Medical University for Nationalities. All subjects signed written informed consent. The diagnosis of PD was based on typical clinical symptoms, signs, and imaging diagnosis. PD patients with tumors, cachexia, severe infections of the system or central nervous system, etc., were excluded. This study was approved by the Ethics Committee of the Affiliated Hospital of Youjiang Medical University for Nationalities (YYFY-LL-2016-04). Under fasting and quiet conditions, the blood of PD patients and healthy individuals were collected, which was centrifuged to collect the supernatant, followed by storage at −80°C.

### RT-qPCR

Total RNA was isolated from blood specimens utilizing an RNeasy mini kit (Qiagen, Germany), and RNA quantity and purity were evaluated using a NanoPhotometer spectrophotometer. For the analysis, 0.2 μg RNA was reverse transcribed utilizing the FastQuant RT Kit (QIAGEN, Germany), followed by adding the SYBR Green master mix (Beyotime, China). Primers for RT-qPCR are as follows: ANGPT2, 5′-AAC​TTT​CGG​AAG​AGC​ATG​GAC-3’ (forward) and 5′-CGA​GTC​ATC​GTA​TTC​GAG​CGG-3’ (reverse); APOD, 5′-ACA​AGC​ATT​TCA​TCT​TGG​GAA​GT-3’ (forward) and 5′-CAT​CAG​CTC​TCA​ACT​CCT​GGT -3’ (reverse); HSP90AA1, 5′-AGG​AGG​TTG​AGA​CGT​TCG​C-3’ (forward) and 5′-AGA​GTT​CGA​TCT​TGT​TTG​TTC​GG-3’ (reverse); HSPA1A, 5′- TGG​TGC​AGT​CCG​ACA​TGA​AG-3’ (forward) and 5′-GCT​GAG​AGT​CGT​TGA​AGT​AGG​C-3’ (reverse); PDE1C, 5′-GAT​GTG​GAC​AAG​TGG​TCC​TTT​G-3’ (forward) and 5′-GGG​GAT​CTT​GAA​ACG​GCT​GA-3’ (reverse); and GAPDH, 5′-CTG​GGC​TAC​ACT​GAG​CAC​C-3’ (forward) and 5′-AAG​TGG​TCG​TTG​AGG​GCA​ATG-3’ (reverse). The relative expression was determined utilizing the 2^−ΔΔCt^ method.

### Western Blotting

The blood specimens were lysed in RIPA buffer, and protein lysate was quantified using a BCA kit (Bio-Rad, United States). Proteins were electrophoresed *via* 8%–12% SDS-polyacrylamide gel and transferred to PVDF membranes. The membranes were probed with the following primary antibodies: ANGPT2 (1:2000; ab155106; Abcam, United States), APOD (1:1,000; ab256496), HSP90AA1 (1:10,000; ab203126), HSPA1A (1:2000; ab194360), PDE1C (1:500; ab14602), and β-actin (1:200; ab115777). Immunoreactive proteins were measured utilizing goat anti-rabbit IgG H&L (HRP)-preabsorbed secondary antibodies (1:2000; ab7090). The protein gray values were quantified by ImageJ software.

### Statistical Analysis

All data were analyzed using R language or GraphPad Prism 8.0.1 software. Two groups were compared with Student’s t-tests. A *p*-value < 0.05 was considered statistically significant.

## Results

### Quality Control and Preprocessing of scRNA-Seq

**FIGURE 1:**
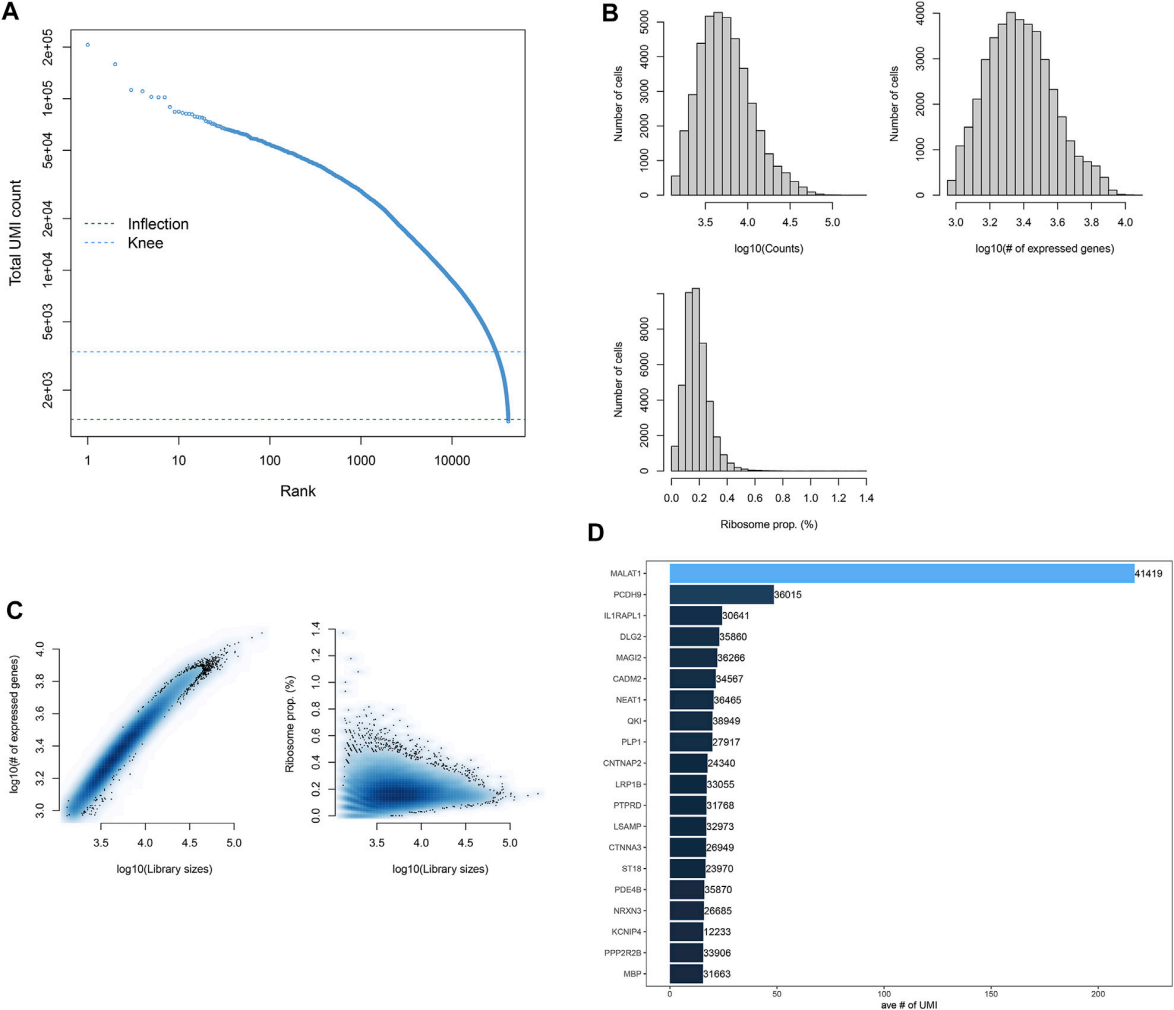
Quality control and preprocessing of scRNA-seq. **(A)** Ranking of cells according to the total UMI count. **(B)** Number of cells that expressed genes. **(C)** Ratio of ribosomal genes expressed in each cell after filtering out cells with <100 expressed genes. **(D)** Top 20 genes expressing more cells.

The scRNA-seq profiles of the midbrain specimens from five PD patients and six healthy individuals were retrieved from the GSE157783 dataset. We counted the expression of each barcode corresponding to each cell and filtered out barcodes without any gene expression (Figure 1A). Figure 1B depicts the distribution of the number of cells according to counts, expressed genes, and ribosome proportions. Cells with the number of expressed genes <100 were filtered out, and then, we counted the ratio of mitochondrial and ribosomal gene expressions in each cell. Figure 1C shows the ratios of the ribosomal gene expression in all cells. We found that the ratios of mitochondrial genes in all cells were zero. Cells with the proportion of ribosomal genes <10% were further removed. Finally, robust and helpful scRNA-seq data were obtained in this study. Figure 1D lists the top 20 gene expressions in more cells, such as *MALAT1*, *PCDH9*, *IL1RAPL1*, *DLG2*, and *MAGI2*.

### Dimensionality Reduction Analysis of scRNA-Seq

**FIGURE 2:**
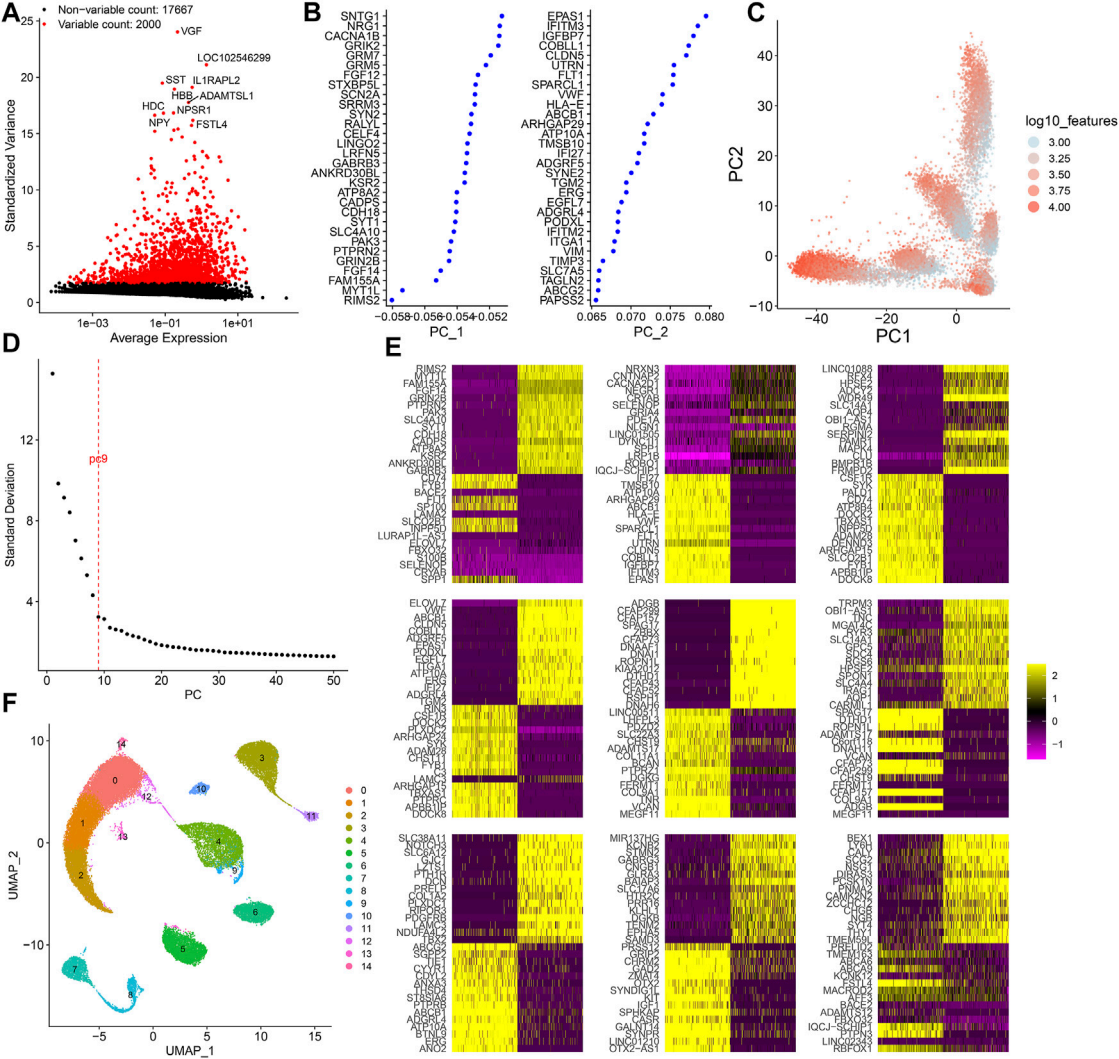
Dimensionality reduction analysis of scRNA-seq. **(A)** Most variable 2000 genes among cells. **(B)** First two PCs of the normalized data. **(C)** Visualization of PCA results in all cells. **(D)** Elbow plot for determining the optimal number of PCs. When PC = 9, the standard deviation was relatively small. **(E)** Heat map for the top 30 feature genes for the first 9 PCs. **(F)** Cell clusters based on UMAP. Each cluster is identified by a unique color.

Following normalization of the filtered scRNA-seq data, we screened the most variable 2000 genes among cells (Figure 2A). Among them, *VGF*, *LOC102546299*, *SST*, *IL1RAPL2*, *HBB*, *ADAMTSL1*, *HDC*, *NPY*, *NPSR1*, and *FSTL4* were most variable. Using the most variable 2000 genes as inputs, PCA results were visualized in order to minimize the number of relevant variables (Figures 2B,C). Figure 2D shows the ranking of the percentage contribution of each PC to the overall variation level. We mainly focused on the PC of the “elbow”. When PC = 9, the standard deviation was relatively small, suggesting that the first nine PCs can better represent the overall change. Figure 2E shows the top 30 marker genes in each PC. To explore the source of heterogeneity, the top 30 feature genes were shown for the first nine PCs. With the UMAP method, cells in the first nine PCs were clustered into 15 categories (Figure 2F).

### Identification of Cell Clusters for PD

**FIGURE 3:**
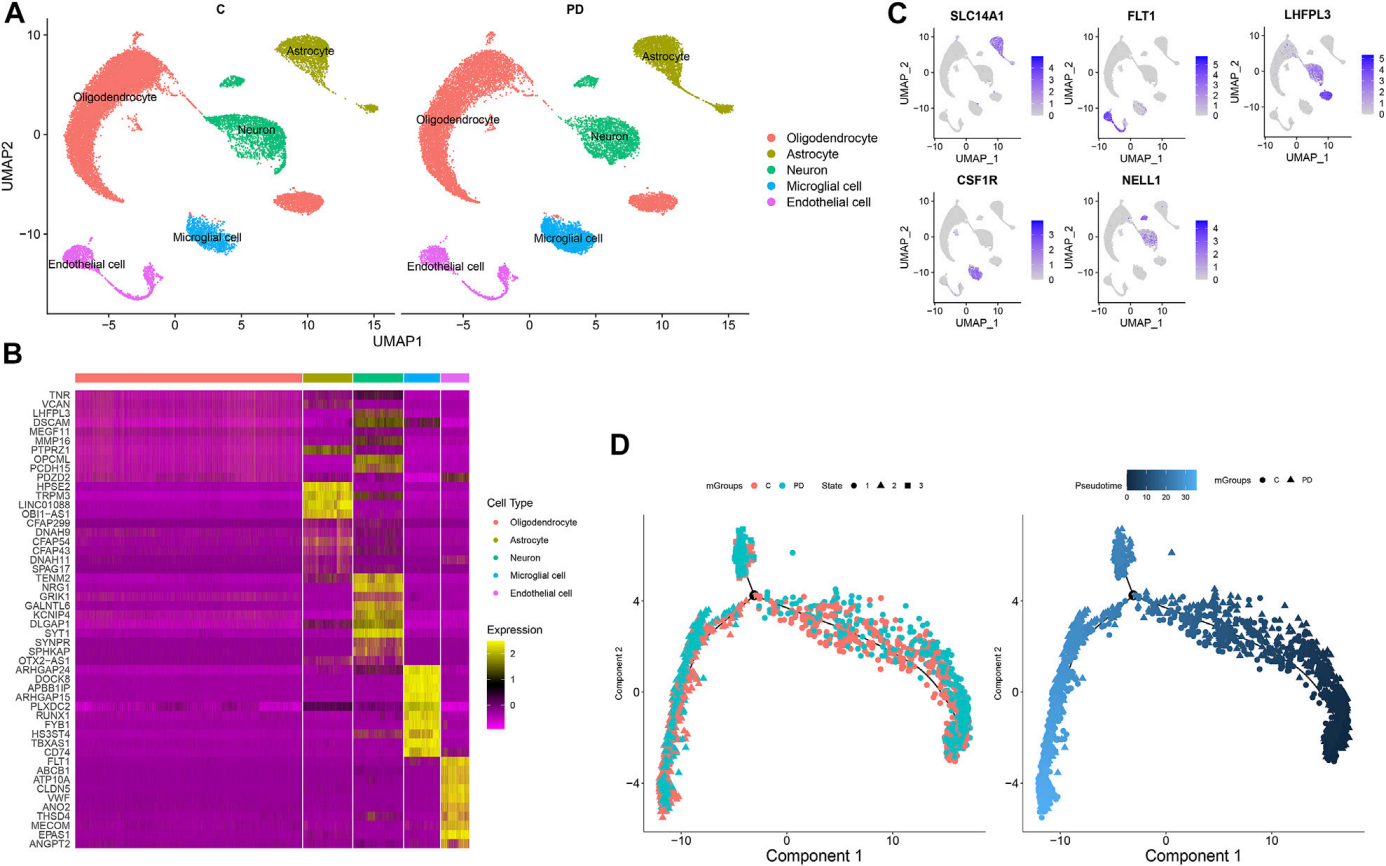
Identification of cell clusters for PD. **(A)** Cell clusters with the UMAP method. **(B)** Heat map for the top 10 marker genes for each cluster. **(C)** Feature genes for each cluster. **(D)** Pseudotime analysis of endothelial cells in PD and healthy samples. Each point represents a single cell.

In accordance with the cell marker genes of the brain in the CellMarker database and the marker genes of each cell cluster, five cell clusters were identified for healthy individuals and PD patients, composed of oligodendrocytes, astrocytes, neurons, microglial cells, and endothelial cells (Figure 3A). Figure 3B shows the differentially expressed genes in each cluster compared with other cell clusters. The gene expression was projected into dimensionality reduction clustering results. As shown in Figure 3C, the top feature genes for oligodendrocytes (*LHFPL3*), astrocytes (*SLC14A1*), neurons (*NELL1* and *LHFPL3*), microglial cells (*CSF1R*), and endothelial cells (*FLT1*) were separately listed. The cell lineage development of endothelial cells was depicted *via* the Monocle 3 package (Figure 3D). The Monocle algorithm was used to learn the dynamic changes in gene expressions experienced by each endothelial cell. PD samples exhibited distinct cell trajectories of endothelial cells compared with healthy individuals’ samples. More PD cells were enriched in branch 3.

### Endothelial Cell as the Core Cell Cluster in Cell–Cell Communications

**FIGURE 4:**
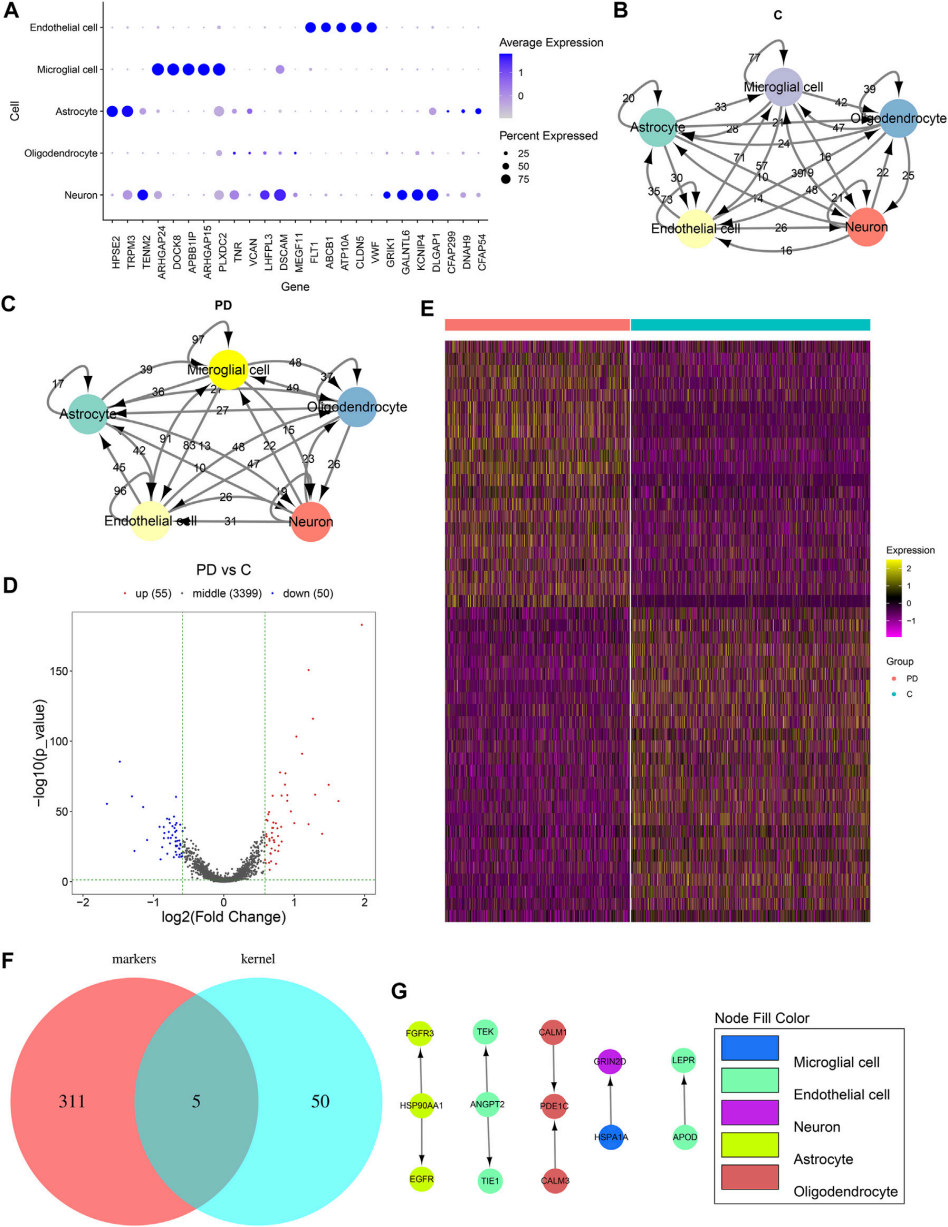
Cell–cell communications across cell clusters. **(A)** Scatter diagram for the top five marker genes of each cell cluster. The size of the dot indicates the percentage of the gene expression in a cell. The color of the dot indicates the expression level of the gene in the cell. Blue indicates high expression. **(B)** Ligand–receptor relationship networks of healthy samples. The arrow points to the recipient cell. The width of the line is proportional to the number of ligand–receptor gene relationships. **(C)** Ligand–receptor relationship networks of PD samples. The arrow points to the recipient cell. The width of the line is proportional to the number of ligand–receptor gene relationships. **(D)** Volcano plot and **(E)** Heat map for the DEGs between PD and healthy endothelial cells. **(F)** Venn diagram for the common genes between upregulated genes and all other marker genes in the cell–cell communication network. **(G)** Relationships between upregulated marker genes. The arrow direction points from the ligand to the receptor.

We selected the top five marker genes of each cell cluster, which are displayed in Figure 4A. The top five marker genes for each cluster are as follows: endothelial cells (*FLT1*, *ABCB1*, *ATP10A*, *CLDN5*, and *VWF*), microglial cells (*ARHGAP24*, *DOCK8*, *APBB1IP*, *ARHGAP15*, and *PLXDC2*), astrocytes (*HPSE2*, *TRPM3*, *CFAP299*, *DNAH9*, and *CFAP54*), oligodendrocytes (*TNR*, *VCAN*, *LHFPL3*, *DSCAM*, and *MEGF11*), and neurons (*TENM2*, *DSCAM*, *GALNTL6*, *KCNIP4*, and *KCNIP1*). Cell to cell communications across different cell clusters depend on associations between ligands and receptors. Herein, we matched the ligand–receptor relationships for the marker genes in each cell cluster. The cell-to-cell communication networks of healthy individuals’ and PD patients’ samples were separately established (Figures 4B,C). We calculated the degree of each cell cluster as follows: endothelial cells (degree = 243), oligodendrocytes (degree = 212), astrocytes (degree = 157), neurons (degree = 81), and microglial cells (degree = 62). In accordance with the cell cluster with the largest number of intercellular receptor–ligand pairs, endothelial cells were identified as the core cell cluster. We extracted the gene expression matrix of endothelial cells. With the cutoff of |FC| > 1.5 and adjusted *p* value <0.05, 55 up- and 50 downregulated genes were identified between PD patients’ and healthy individuals’ endothelial cells (Figure 4D). Supplementary Table S1 lists the 55 upregulated genes. Moreover, Supplementary Table S2shows the 50 downregulated genes. A heat map depicted that these DEGs could distinguish PD patients from healthy individuals (Figure 4E). We further intersected the upregulated genes with all other marker genes in the cell–cell communication network. As a result, five upregulated marker genes were identified, including *ANGPT2*, *APOD*, *HSP90AA1*, *HSPA1A*, and *PDE1C* (Figure 4F). Figure 4G shows their ligand–receptor interactions in all cell clusters. In endothelial cells, ANGPT2-TEK, ANGPT2-TIE1, and APOD-LEPR relationships were found.

### Potential Biological Functions of Differentially Expressed Genes (DEGs) Between PD Patients’ and Healthy Individuals’ Endothelial Cells

**FIGURE 5:**
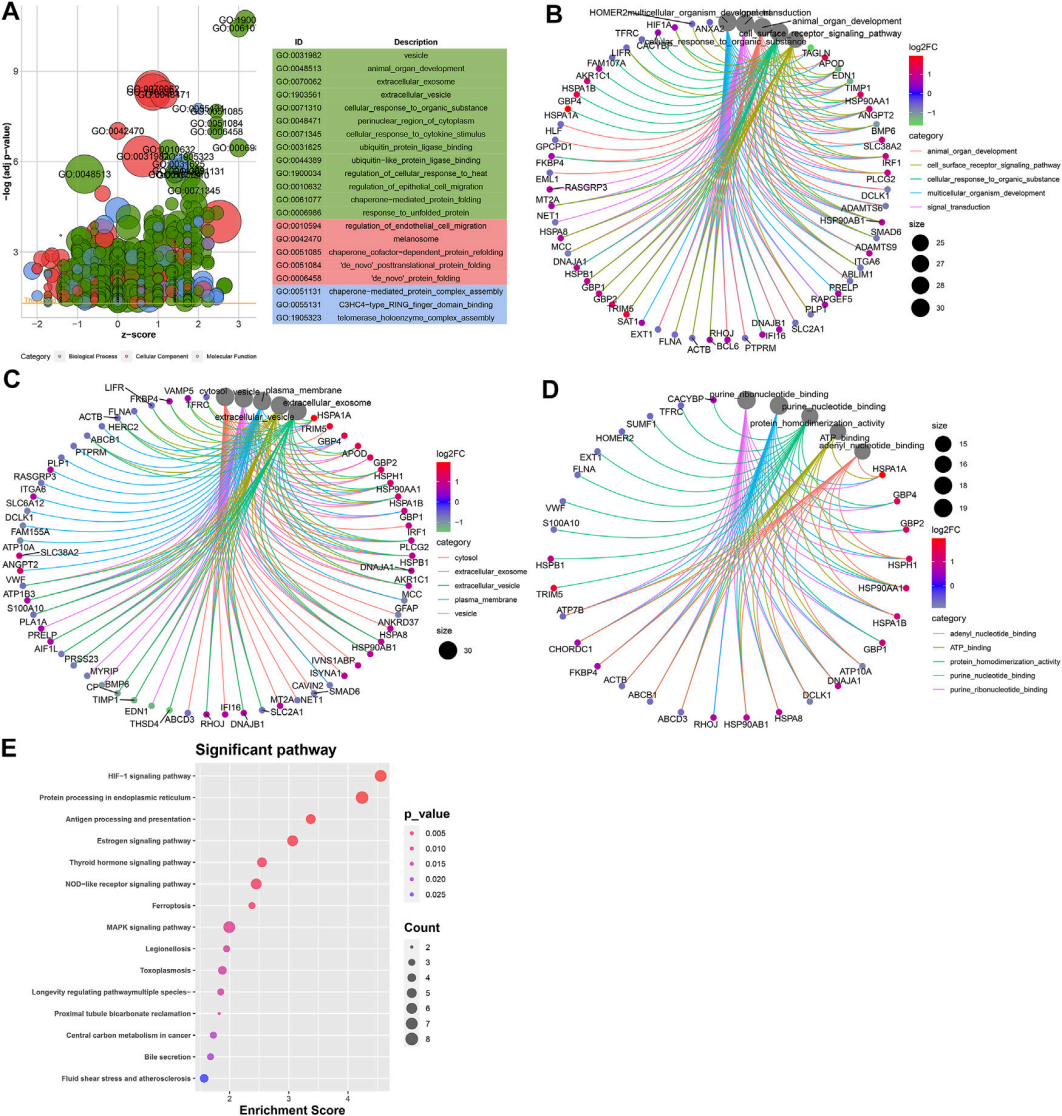
Potential biological functions of DEGs in endothelial cells. **(A)** Bubble chart for the top 20 GO enrichment results including the biological process (BP; green), cellular component (CC; red), and molecular function (MF; blue). Core networks for the top five **(B)** BP, **(C)** CC, and **(D)** MF terms and enriched genes. **(E)** Heat map for the top 15 KEGG pathways and enriched genes. The cell color represents the |log2FC| of genes. The warm color indicates upregulated genes, and the cool color indicates downregulated genes.

To probe the underlying biological functions of DEGs in endothelial cells, we presented the GO enrichment analysis. Figure 5A depicts the top 20 GO terms enriched by DEGs. These DEGs were involved in animal organ development, cell surface receptor signaling pathway, cellular response to an organic substance, multicellular organism development, and signaling transduction biological processes (Figure 5B). In Figure 5C, they participated in cellular components of cytosol, extracellular exosome, extracellular vesicle, plasma membrane, and vesicle. Furthermore, they had the molecular functions of adenyl nucleotide–binding, ATP-binding, protein homodimerization, purine nucleotide–binding, and purine ribonucleotide–binding (Figure 5D) activities. We further analyzed the KEGG pathways enriched by these DEGs. In Figure 5E, these DEGs were enriched in HIF-1, protein processing in the endoplasmic reticulum, MAPK, NOD-like receptor, antigen processing and presentation, GABAergic synapse, and Ras and Rap1 signaling pathways.

### Hub Genes in Endothelial Cells

**FIGURE 6:**
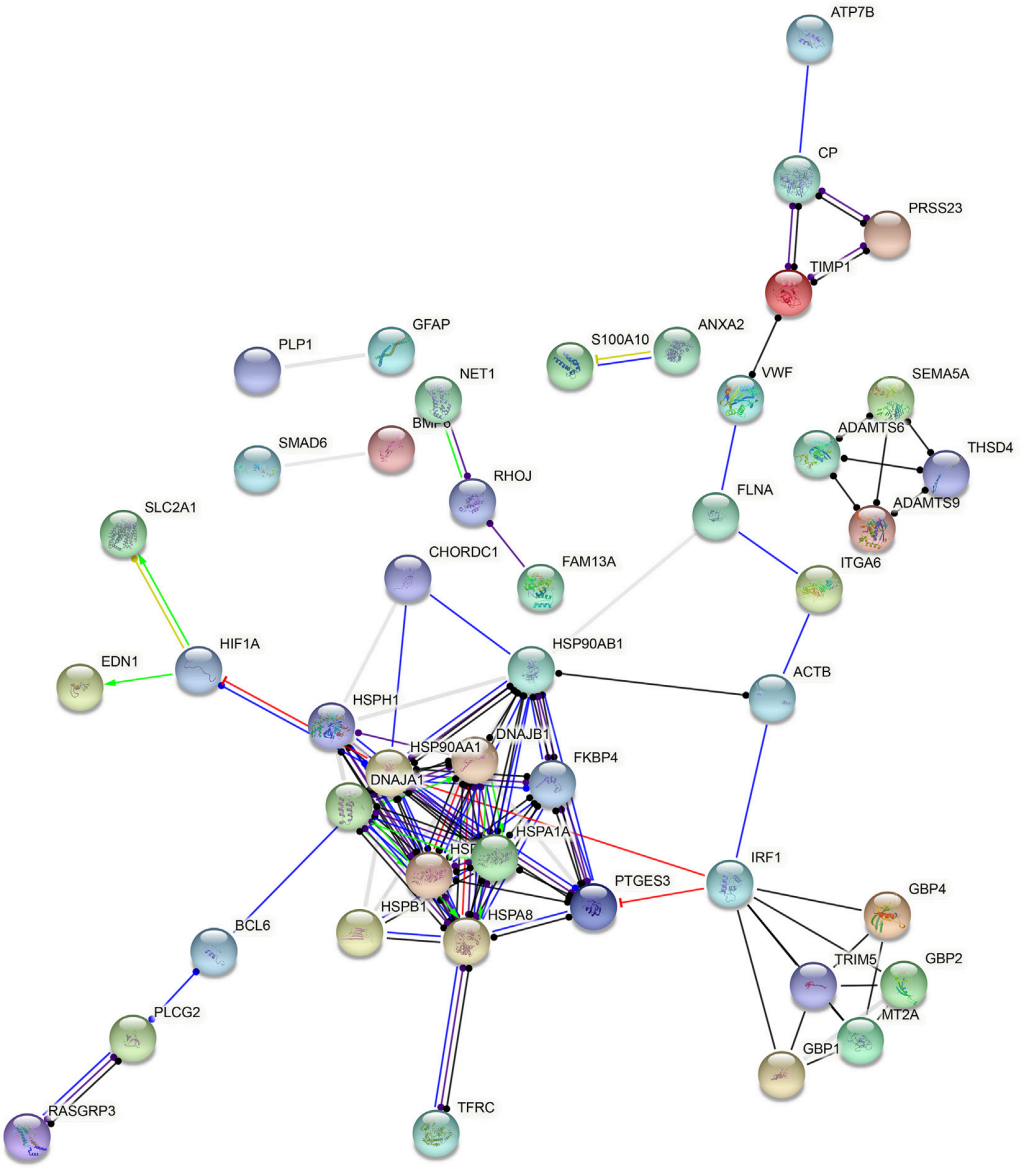
Hub genes in endothelial cells. A PPI network was constructed based on all DEGs in endothelial cells.

Based on all DEGs in endothelial cells, a PPI network was constructed, composed of 48 nodes (Figure 6). The top ten nodes with the highest degree were considered as hub genes, including HSP90AA1 (degree = 14), HSP90AB1 (degree = 13), HSPA8 (degree = 11), HSPA1A (degree = 10), DNAJB1 (degree = 9), HSPA1B (degree = 9), HSPH1 (degree = 8), IRF1 (degree = 8), PTGES3 (degree = 8), and DNAJA1 (degree = 7).

### Validation of Marker Genes in the Cell–Cell Communication Network

**FIGURE 7:**
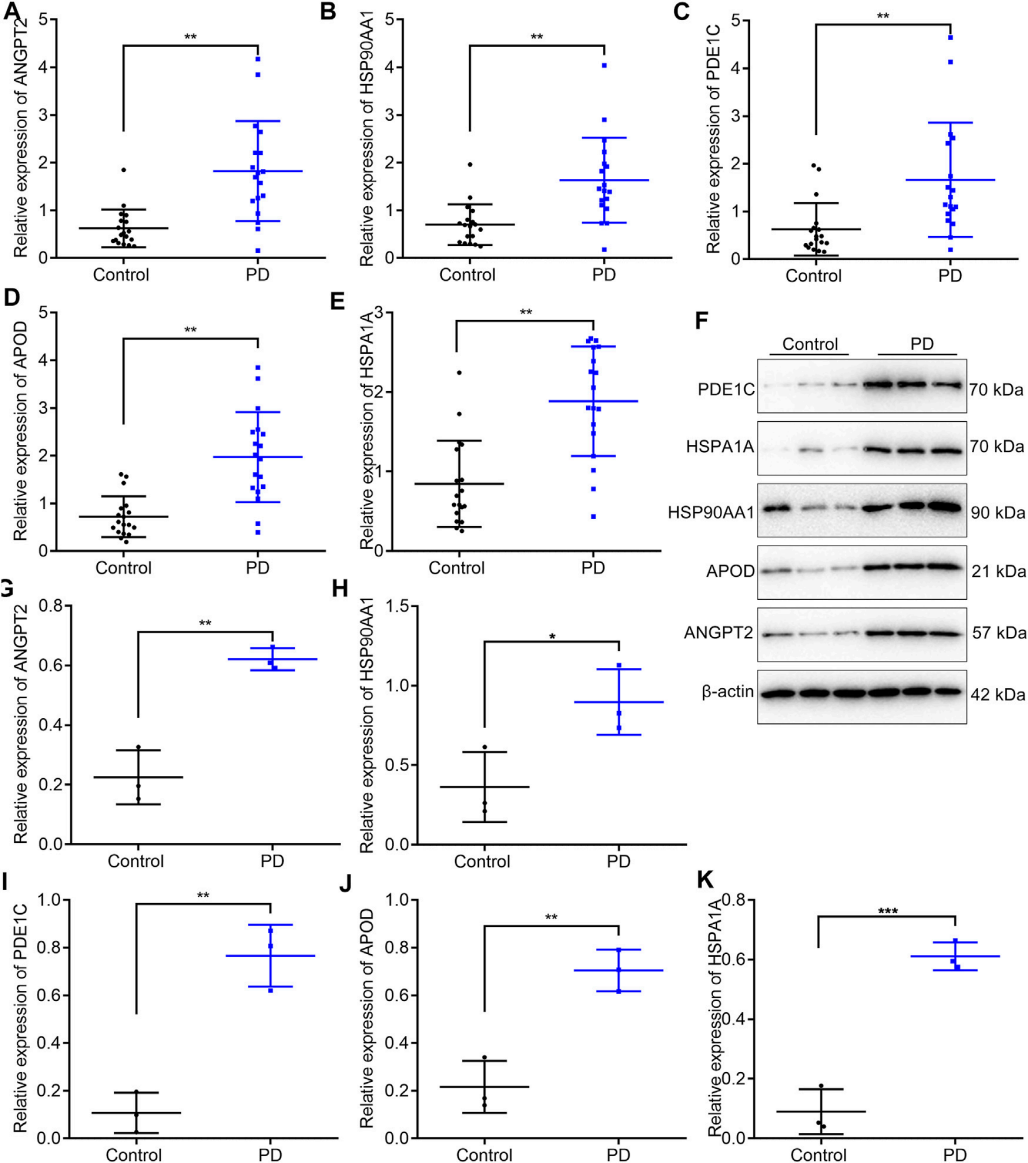
Validation of the expression of five upregulated marker genes in the cell–cell communication network in blood samples from PD patients and healthy participants. **(A–E)** RT-qPCR for detecting the mRNA expressions of *ANGPT2*, *HSP90AA1*, *PDE1C*, *APOD*, and *HSPA1A* in the blood specimens from 18 PD patients and 18 healthy participants. **(F–K)** Western blotting for measuring the protein expressions of ANGPT2, HSP90AA1, PDE1C, APOD, and HSPA1A in the blood specimens from 18 PD patients and 18 healthy participants. *p < 0.05; **p < 0.01; and ***p < 0.001.

In total, 18 PD patients and 18 healthy participants were enrolled in our study. Five upregulated marker genes in the cell–cell communication network were detected in blood samples through RT-qPCR and Western blotting. RT-qPCR results confirmed that ANGPT2 (Figure 7A), HSP90AA1 (Figure 7B), PDE1C (Figure 7C), APOD (Figure 7D), and HSPA1A (Figure 7E) mRNAs were markedly upregulated in blood specimens from PD patients compared with healthy participants. Their high expressions in PD blood samples were also confirmed by Western blotting (Figures 7F–K).

## Discussion

In this study, we comprehensively analyzed the cellular heterogeneity of PD *via* single-cell transcriptomics. In the cell–cell communications, endothelial cells were the core cell cluster. The DEGs in endothelial cells possessed key biological functions. Five upregulated marker genes were identified for endothelial cells, which could become promising therapeutic targets for PD.

Cell heterogeneity is a common feature of biological systems and biological tissues. Single-cell sequencing technology reveals the genetic differences between different cell populations, which is conducive to the refinement of cell classification and expansion of the tree of life. Herein, we employed scRNA-seq data of the midbrain specimens from PD patients and healthy individuals. In the previous research, 12 cell types were identified including astrocytes, CADPS2^high^, DaNs, endothelial cells, ependymal cells, excitatory cells, GABA, inhibitory cells, microglial cells, OPCs, oligodendrocytes, and pericytes (Badanjak et al., 2021). Different from previous research, five cell clusters were identified in this study, composed of oligodendrocytes, astrocytes, neurons, microglial cells, and endothelial cells. Cells are the basic unit of organisms. Even if they are derived from the same individual and the same cell line, specific cells may have different characteristics in the genome and transcriptome. Previous genomic research results have often shown the average value of gene expressions in a group of cells, so it is difficult to clarify the specific cell types that play a key role in the development of life and disease. With the rapid development of molecular biology techniques, the whole genome or transcriptome of a single cell is amplified and then sequenced by the next-generation sequencing technology. scRNA realizes single-cell whole-genome sequencing, thereby revealing differences in cell populations and evolution (Picelli, 2017; Rostom et al., 2017; Papalexi and Satija, 2018). Our data suggested that endothelial cells could play a major role in the cell–cell communication for PD. Endothelial cells may regulate the blood–brain barrier permeability, interactions between cells and matrix, and angiogenesis and the like (Sweeney et al., 2019). Five marker genes were upregulated in PD patients’ than in healthy individuals’ endothelial cells, including *ANGPT2*, *APOD*, *HSP90AA1*, *HSPA1A*, and *PDE1C*. Their upregulation was confirmed in 18 PD patients’ compared with 18 healthy individuals’ blood specimens through RT-qPCR and Western blotting. Among them, APOD, an anti-inflammatory and antioxidant lipocalin transporter of the small hydrophobic molecule, is widely expressed in the brain tissues and plasma. A cross-sectional study found that serum ApoD levels had a significant correlation with the PD stage (Waldner et al., 2018). It has been confirmed that upregulation of ApoD possesses a neuroprotective function (Dassati et al., 2020). HSPA1A, a molecular chaperone, can prevent and decelerate PD-like neurodegeneration (Ekimova et al., 2018). Combining previous studies, these five upregulated marker genes deserve in-depth clinical research.

We found that DEGs in endothelial cells participated in organism development such as animal organ development, cell surface receptor signaling pathway, cellular response to an organic substance, multicellular organism development, and signaling transduction biological processes. Furthermore, they were involved in PD-related cellular components such as cytosol, extracellular exosome, extracellular vesicle, plasma membrane, and vesicle. Extracellular vesicle includes exosome and microvesicle, which may remove harmful molecules and spread PD-related pathogenic proteins (Hill, 2019). For example, extracellular vesicles secreted from neural cells may mediate homeostasis of the brain and communications between neural and peripheral cells (Upadhya et al., 2020). Our data also showed that DEGs in endothelial cells had the molecular functions of adenyl nucleotide–binding, ATP-binding, protein homodimerization activity, purine nucleotide–binding, and purine ribonucleotide–binding activities. The KEGG pathway enrichment analysis demonstrated that these DEGs were enriched in various key pathways such as HIF-1, protein processing in the endoplasmic reticulum, MAPK, NOD-like receptor, antigen processing and presentation, GABAergic synapse, and Ras and Rap1 signaling pathways (Li et al., 2019). These data highlighted the critical roles of DEGs in endothelial cells.

Immune-related pathways such as primary immunodeficiency, antigen processing and presentation, natural killer cell–mediated cytotoxicity, T-cell receptor, and B-cell receptor were overexpressed in PD patients’ samples than in healthy individuals’ samples. Excessive inflammatory responses in brain tissues may lead to neurodegeneration and parkinsonism (Grozdanov et al., 2019). Immunotherapy has been developed for treating PD in recent years. However, appropriate immune-related markers are still lacking (Williams-Gray et al., 2016). Furthermore, the purine metabolism, VEGF, chemokine, JAK/STAT, toll-like, MAPK, and calcium signaling pathways were overexpressed in PD patients’ than in healthy individuals’ endothelial cells. Based on these DEGs in endothelial cells, we established a PPI network and identified ten hub genes, including *HSP90AA1*, *HSP90AB1*, *HSPA8*, *HSPA1A*, *DNAJB1*, *HSPA1B*, *HSPH1*, *IRF1*, *PTGES3*, and *DNAJA1*. Among them, upregulated *HSP90AB1* exerts complementary effects on protein misfolding during PD (Xie et al., 2016). IRF1 upregulation facilitates the cerebral vascular endothelial inflammatory response in PD (Yunfu et al., 2014). The roles of hub genes in PD progression require more exploration.

## Conclusion

In this study, we characterized five cell clusters including oligodendrocytes, astrocytes, neurons, microglial cells, and endothelial cells. Among them, endothelial cells were the core cell cluster in cell–cell communications. Furthermore, five marker genes were upregulated in PD than in healthy endothelial cells, which could be underlying therapeutic targets for PD. DEGs in endothelial cells participated in various biological functions, highlighting the key roles of endothelial cells in PD.

## Data Availability

The original contributions presented in the study are included in the article/Supplementary Material, further inquiries can be directed to the corresponding author.

## Abbreviations

PD, Parkinson’s disease; scRNA-seq, single-cell RNA sequencing; UMAP, Uniform Manifold Approximation and Projection for Dimension Reduction; GEO, Gene Expression Omnibus; UMI, unique molecular identifier; PCA, principal component analysis; PCs, principal components; FC, fold change; DEGs, differentially expressed genes; DAVID, Database for Annotation Visualization and Integrated Discovery; BP, biological process; CC, cellular component; MF, molecular function; KEGG, Kyoto Encyclopedia of Genes and Genomes; PPI, protein–protein interaction.
